# Development of new fusion proteins for visualizing amyloid-β oligomers *in vivo*

**DOI:** 10.1038/srep22712

**Published:** 2016-03-16

**Authors:** Tomoyo Ochiishi, Motomichi Doi, Kazuhiko Yamasaki, Keiko Hirose, Akira Kitamura, Takao Urabe, Nobutaka Hattori, Masataka Kinjo, Tatsuhiko Ebihara, Hideki Shimura

**Affiliations:** 1 Biomedical Research Institute, National Institute of Advanced Industrial Science and Technology (AIST), 1-1-1, Higashi, Tsukuba, Ibaraki 305-8566, Japan; 2 Laboratory of Molecular Cell Dynamics, Faculty of Advanced Life Science, Hokkaido University, N21W11, Kita-ku, Sapporo 001-0021, Japan; 3 Department of Neurology, Juntendo University Urayasu Hospital, 2-1-1, Tomioka, Urayasu, Chiba 279-0021, Japan; 4 Department of Neurology, Juntendo University School of Medicine, 2-1-1, Hongo, Bunkyo-ku, Tokyo 113-8421, Japan

## Abstract

The intracellular accumulation of amyloid-β (Aβ) oligomers critically contributes to disease progression in Alzheimer’s disease (AD) and can be the potential target of AD therapy. Direct observation of molecular dynamics of Aβ oligomers *in vivo* is key for drug discovery research, however, it has been challenging because Aβ aggregation inhibits the fluorescence from fusion proteins. Here, we developed Aβ_1-42_-GFP fusion proteins that are oligomerized and visualize their dynamics inside cells even when aggregated. We examined the aggregation states of Aβ-GFP fusion proteins using several methods and confirmed that they did not assemble into fibrils, but instead formed oligomers *in vitro* and in live cells. By arranging the length of the liker between Aβ and GFP, we generated two fusion proteins with “a long-linker” and “a short-linker”, and revealed that the aggregation property of fusion proteins can be evaluated by measuring fluorescence intensities using rat primary culture neurons transfected with Aβ-GFP plasmids and Aβ-GFP transgenic *C. elegans*. We found that Aβ-GFP fusion proteins induced cell death in COS7 cells. These results suggested that novel Aβ-GFP fusion proteins could be utilized for studying the physiological functions of Aβ oligomers in living cells and animals, and for drug screening by analyzing Aβ toxicity.

Alzheimer’s disease (AD) is a neurodegenerative disease characterized by the progressive loss of cognitive functions. A typical neuropathological features of AD is the deposition of senile plaques that are composed of the fibrillar amyloid β (Aβ) protein^1^,^2^. Although extracellular Aβ deposition is well documented, emerging evidence indicates that Aβ also accumulates intraneuronally and might be critically involved in the progression of cognitive decline^3^. For example, intraneuronal accumulations of Aβ reduce the expression of synaptic proteins^4^, contribute to tau phosphorylation^5^, and mitochondrial dysfunction^6^,^7^. Therefore the physiological functions of intraneuronal Aβ in non-fibrillar or water soluble forms have attracted increasing attention, and numerous reports have provided extensive evidence indicating that low molecular weight Aβ oligomers may act as the key molecule of the synaptic disorder^8^,^9^,^10^,^11^,^12^,^13^,^14^,^15^. The amyloid precursor protein (APP) E693Δ mutant causes AD in humans. Expression of this mutant in mice resulted in age dependent accumulation of intraneuronal Aβ oligomers without formation of extracellular amyloid deposits, and induced synaptic and neuronal losses^16^. However, despite insights provided by biochemical, genetic, and animal model studies, effective therapeutic drugs that treat the symptoms of AD have not been developed. Direct observation of the process of accumulation and disaggregation of intracellular Aβ oligomers *in vivo* is critical for evaluating the efficiency of candidate therapeutic molecules and investigating the function of Aβ.

However, a major technical challenge is that it has been difficult to visualize Aβ in living cells when fused to the fluorescent proteins, such as GFP. Formation of the chromophore of fluorescent proteins depends on correct folding of the protein, and insoluble aggregation of the fused protein tends to cause loss of fluorescence^17^. Therefore, C-terminal fusion proteins containing wild type Aβ_1-42_ joined to GFP normally does not fluoresce, probably because Aβ_1-42_ aggregation results in GFP misfolding. Mutagenesis in the hydrophobic region of Aβ_1-42_, which contains the determinants of Aβ_1-42_ aggregation, reduced the insolubility and enabled detectable fluorescence of an Aβ_1-42_ -GFP mutant^18^.

In the current study, we tried to visualize the molecular dynamics of wild type Aβ_1-42_ *in vivo* by arranging the length of linker sequence between Aβ_1-42_ and GFP in Aβ-GFP fusion proteins. Using this fusion protein, we revealed that Aβ_1-42_-GFP formed oligomers both *in vivo* and *in vitro*. The fusion proteins developed in this study are useful tools for screening candidate molecules of therapeutic drugs of AD and for investigating the function of intracellular oligomeric Aβ_1-42_ in cells.

## Results

### Visualizing Aβ-GFP fusion proteins in COS7 cells

**Figure 1:**
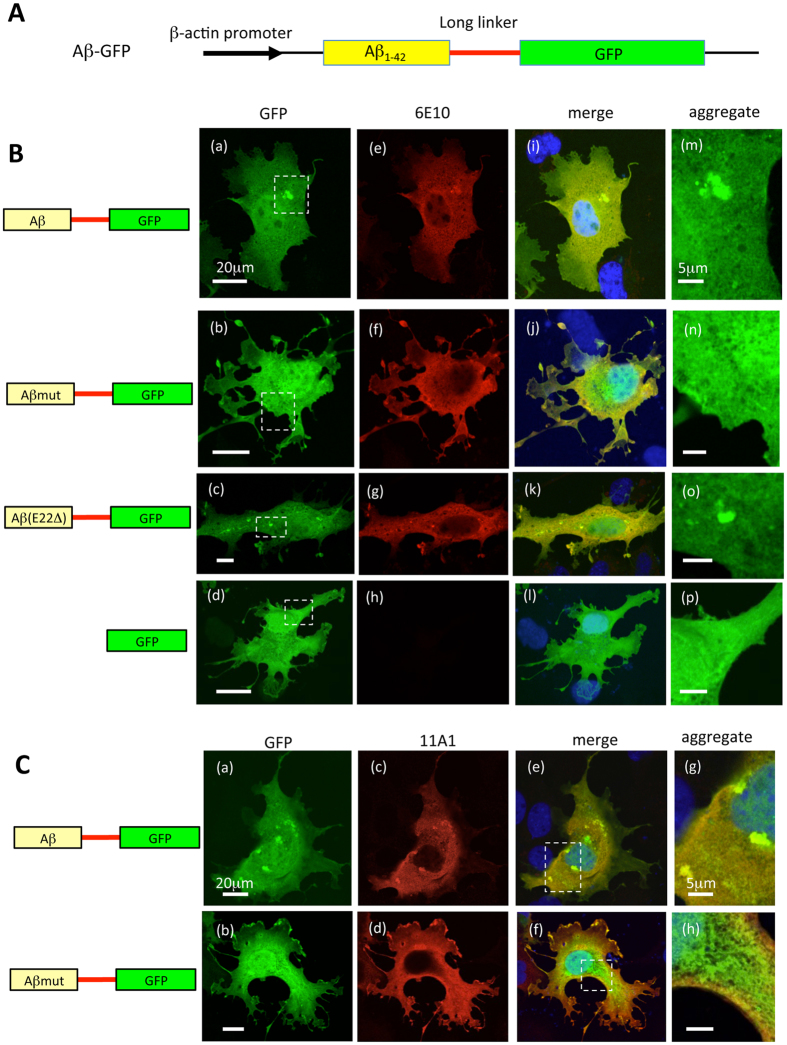
Representative images of COS7 cells transfected with various Aβ-GFP DNA constructs. (**A**) Basic structure of genes encoding fusion protein containing Aβ_1-42_ fused to GFP with a long-linker sequence (14 amino acids). (**B**) COS7 cells were transfected with plasmids encoding Aβ-GFP (a) Aβmut-GFP (b) Aβ (E22Δ)-GFP (c), or GFP (d). To confirm the expression of Aβ proteins, transfected cells were immunostained with the 6E10 antibody (e–h). Merged images with GFP are shown in (i–l). The regions within the dotted rectangles in (a–d) are enlarged in (m–p). Aggregated Aβ proteins (dotted localizations) were observed in Aβ-GFP and Aβ (E22Δ)-GFP transfected cells, however, the Aβmut-GFP proteins did not form detectable aggregates in cells. Scale bars: 20 μm (a–d) 5 μm (m–p). (**C**) Immunostaining of COS7 cells expressing the Aβ-GFP or Aβmut-GFP fusion proteins with the 11A1 antibody. Merged images showed that almost all the Aβ-GFP fusion protein was labeled with the11A1 antibody, indicating that the Aβ-GFP fusion protein formed oligomers. In contrast, the Aβmut-GFP was only partially labeled with the11A1 antibody. Scale bars: 20 μm (a–f) 5 μm (g,h).

Previous Aβ mutagenesis studies showed that the C-terminal fusion of Aβ_1-42_ to GFP prevents exact folding of the GFP protein in *Escherichia coli (E. coli)*, thereby GFP does not fluoresce, whereas GFP fusion with a non-aggregating variant of Aβ_1-42_ showed retained GFP fluorescence^18^. To visualize the molecular dynamics of wild type Aβ_1-42_, we developed a new GFP fusion construct that fluoresces even when the fused Aβ_1-42_ proteins are aggregated. This construct, which derives the expression of human Aβ_1-42_ fused to the N-terminus of GFP, encodes a long linker sequence of 14 amino acids (QSTVPRARDPPVAT) between Aβ and GFP (Fig. 1A).

To observe the expression patterns of various Aβ-GFP fusion proteins, COS7 cells were transfected with the plasmids encoding Aβ-GFP (Fig. 1Ba), Aβmut-GFP (Fig. 1Bb), or Aβ (E22Δ)-GFP (Aβ bearing a mutation causing Alzheimer’s disease; Osaka mutation, Fig. 1Bc). The Aβmut-GFP protein contains F19S and L34P substitution, which were reported to suppress the aggregation of Aβ_1-42_^18^. In addition, COS7 cells were transfected with a GFP construct alone as a control (Fig. 1Bd). To confirm the expression of the Aβ proteins, transfected cells were immunostained with an anti- β amyloid antibody (6E10; Fig. 1Be–h), which recognizes all species of Aβ, i.e., monomer, oligomer, and fibril forms of it^19^. As shown in Fig. 1Bi–k, almost all GFP fluorescence in the cytoplasm of transfectants were colocalized with fluorescence of 6E10 antibody, except for cells transfected with GFP alone (Fig. 1Bl), indicating that GFP signals coincide with the localization sites of each Aβ fusion protein.

Cells transfected with GFP alone showed almost uniform GFP expression in the cytoplasm and nucleus (Fig. 1Bd,p), as also observed with Aβmut-GFP transfected cells (Fig. 1Bb,n). In contrast, cells expressing the Aβ-GFP fusion protein showed aggregates of various sizes and shapes of Aβ-GFP in the cytoplasm (Fig. 1Ba,m), although the nuclear distribution appeared uniform. The expression patterns of the Aβ(E22Δ)-GFP fusion protein were similar to that of the Aβ-GFP fusion protein, as aggregates of various sizes and shapes of Aβ(E22Δ)-GFP were also observed throughout the cells (Fig. 1Bc,o).

To assess the polymerization states of Aβ-GFP fusion proteins inside of cells, each transfectant was immunostained using the 11A1 antibody, which was developed against E22P-Aβ_10-35_ as an antigen and recognizes oligomeric forms of Aβ specifically^20^. Almost all GFP signals were double-labeled by the 11A1 antibody in Aβ-GFP transfected cells (Fig. 1C), suggesting that the Aβ-GFP aggregates were oligomer (Fig. 1Cg). However, the Aβmut-GFP fusion proteins were only partially double-labeled with the 11A1 antibody, especially in the peripheral regions of the cell (Fig. 1Ch), indicating that most of the Aβmut-GFP proteins do not form oligomers inside of cells. Immunoblot analysis following native-PAGE also supports the results of immunostaining. Non-denaturing protein lysates from COS7 cells that expressed each Aβ-GFP fusion protein were separated on a gel. We used anti-GFP and anti-Aβ (6E10) antibodies to detect the fusion proteins. A clear single band close to the GFP signal was detected in Aβmut-GFP lysate by both antibodies. In contrast, smear and ladder-like signals were observed in both Aβ-GFP and Aβ (E22Δ)-GFP lysates by these antibodies. These results indicated that most of the Aβmut-GFP proteins exist as small-sizes molecules, probably monomers but Aβ-GFP and Aβ (E22Δ)-GFP proteins exist as oligomers of different sizes in the cell (see Supplementary Fig. S1 and methods online).

We investigated the specific subcellular localization site of the Aβ-GFP fusion protein by double labeling with antibodies against marker proteins specific for mitochondria, Golgi apparatus, or endoplasmic reticulum, however, no double labeling was detected in those intracellular organelles (data not shown). To observe when and how the fusion proteins are expressed and accumulated in cells, we performed the time-laps imaging of COS7 cells transiently expressing Aβ-GFP (Supplementary Fig. S2). The Aβ-GFP fusion protein gradually aggregated in a time-dependent manner.

### Comparison of GFP fluorescence intensity of Aβ-GFP fusion proteins according to the length of the linker sequence

**Figure 2:**
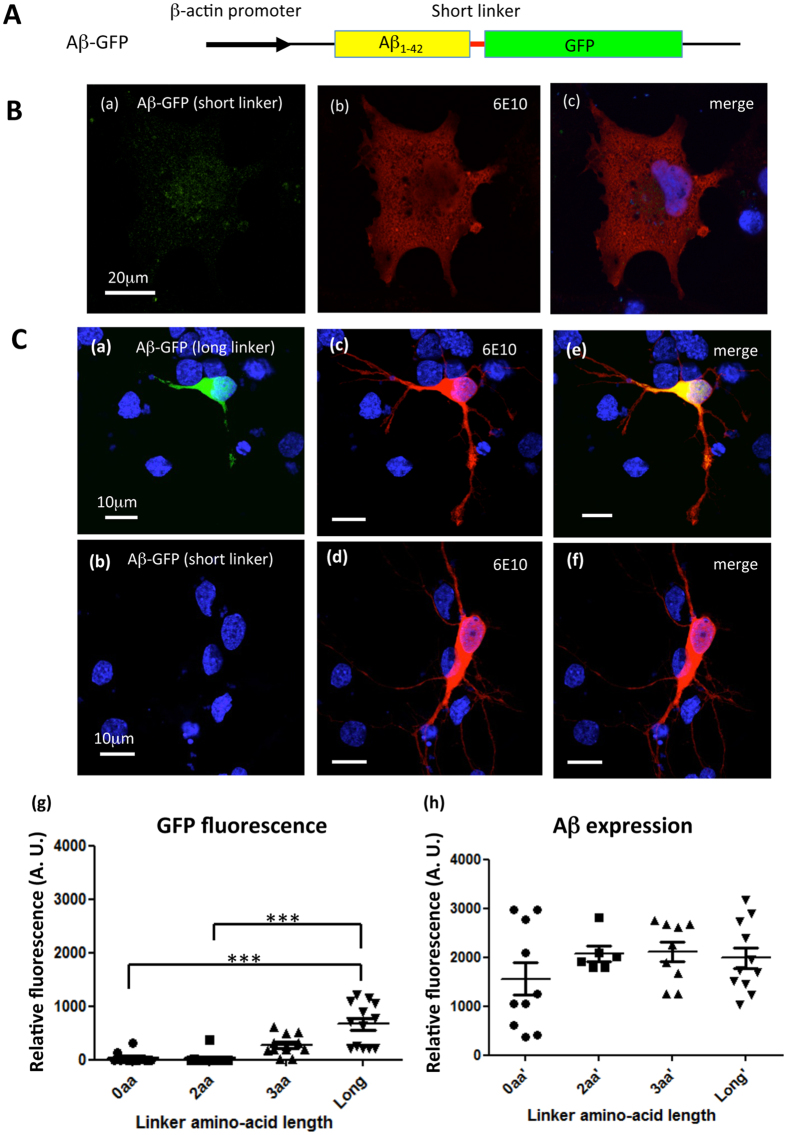
Comparison of Aβ-GFP fluorescence intensities according to the linker length in primary culture neurons. (**A**) Basic structure of genes encoding fusion proteins containing Aβ_1-42_ fused to GFP with short-linker sequences (0, 2 or 3 amino acids). (**B**) COS7 cells transfected with a short-linker Aβ-GFP (2 amino acids). Faint GFP fluorescence was detected in the nucleus and surrounding areas (a) even though the fusion protein was stained by the 6E10 antibody (b). Merged image of (a,b) is shown in (c). Scale bar: 20 μm. (**C**) Primary culture of rat hippocampal neurons transfected with Aβ-GFP plasmids containing long-linker (a) or short-linkers (b). GFP fluorescence was nearly undetectable in cells carrying the short-linker plasmids, even though the fusion protein was stained by the 6E10 antibody (c,d). Merged images with GFP are shown in (e,f). Relative fluorescence intensities from cells expressing each fusion protein with various linker lengths were measured (g,h). Statistical analyses showed that the detection of the Aβ protein in neurons was nearly identical with each plasmid (h) but GFP fluorescence intensities increased significantly as the linker became longer (g) (***p < 0.001, Kruskal-Wallis test, n = 10–14 cells each). Scale bar: 10 μm.

We considered that proper folding of GFP in fusion proteins may depend on the linker length between Aβ and GFP, and that the folding efficacy may affect the fluorescent intensity. To determine the effect of the linker length on the fluorescence intensities of fusion proteins, Aβ-GFP plasmids with a short-linker (0, 2, or 3 amino acid) or a long-linker (14 amino acids) were transfected into COS7 cells and rat hippocampal primary culture neurons, and the fluorescence intensities of the GFP fusion proteins were compared. Figure 2 shows images of cells expressing fusion proteins with a 2-amino acid linker (short-linker) or a long-linker. Twenty-four hours after transfection, both COS7 cells and primary neurons were immunolabeled by the 6E10 antibody, and confocal images were taken under the completely same condition as described in the “Methods” section. GFP fluorescence showed uniform cytoplasmic distribution in both cell types transfected with the long-linker construct, (Figs 1Ba and 2Ca). However, in cells transfected with the short-linker construct, GFP fluorescence was undetectable in the cytoplasm and was very faint in the nucleus (Fig. 2Ba,Cb), even though the immunolabeling signals of the 6E10 antibody were detected strongly (Fig. 2Bb,Cd). Comparison of the staining intensities observed with the 6E10 antibody and that of GFP fluorescence was performed in neurons expressing Aβ-GFP proteins with differing linker length (Fig. 2Cg,h). The immunofluorescence intensities observed with 6E10 were nearly identical for each fusion protein, but the GFP fluorescence intensities decreased as the length of the linker became shorter. These results indicated that GFP fused to Aβ via long-linker can fold normally and fluorescence robustly, whereas GFP cannot fluorescence robustly in the short-linker forms, probably because of misfolding.

### Oligomerization of the Aβ-GFP fusion protein *in vitro*

**Figure 3:**
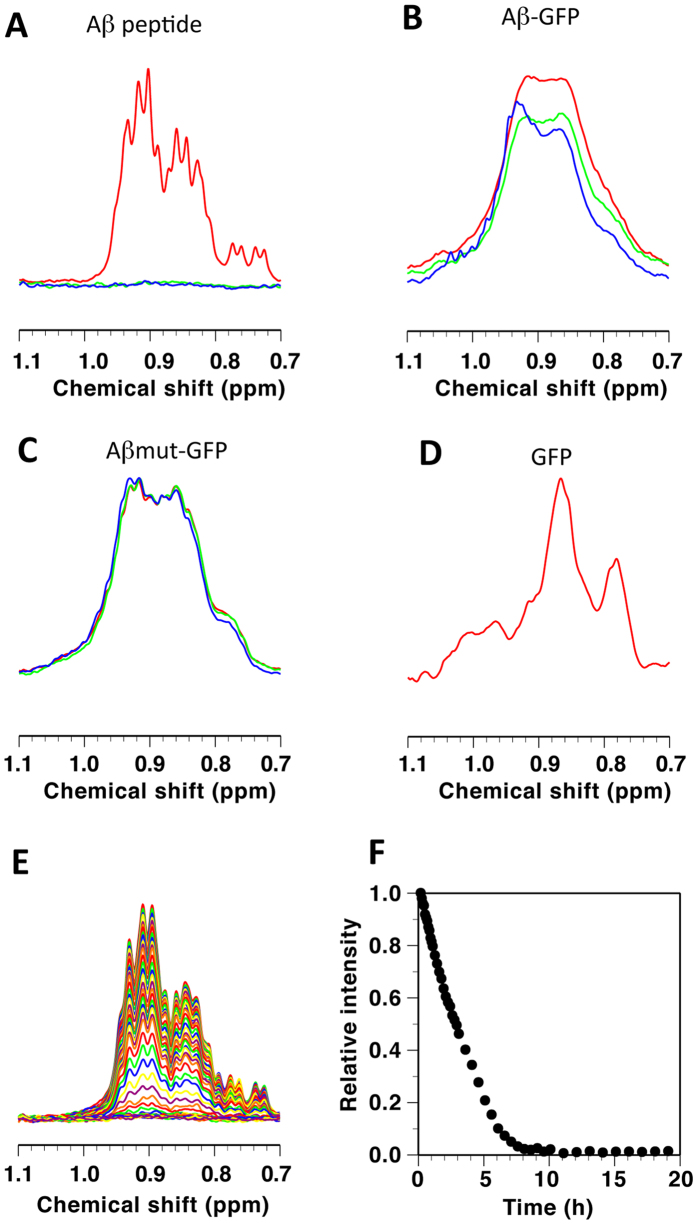
NMR analyses of structural changes in Aβ-GFP fusion proteins. Shown are parts of 500-MHz NMR spectra mainly reflecting methyl groups for the Aβ peptide (**A,E**), Aβ-GFP (**B**), Aβmut-GFP (**C**), and GFP (**D**). Spectra in (**A–D**) were recorded at 20 °C, where the red, green and blue lines indicate intact peptides, those after incubation at 37 °C for 15.5 h, and those after incubation at 37 °C for 50 h or 63.5 h, respectively. Spectra in (**E**) were recorded at 37 °C after 5 min −19 h of incubation, where changes in the intensity of the highest peak at 0.91 ppm are shown in (**F**).

To analyze the molecular characteristics of the Aβ-GFP fusion proteins in detail, we performed nuclear magnetic resonance (NMR) measurements and electron microscopy (EM) observations. We focused on the long-linker fusion proteins because only these proteins appeared to be folded normally. NMR spectra of synthetic peptide (Fig. 3A,E,F), Aβ-GFP (B), Aβmut-GFP (C) and GFP (D) were collected to examine aggregation. We used Hou’s method^21^ to generate the monomeric Aβ fusion proteins, as described in the “Methods” section. The NMR spectral intensities of the synthetic Aβ peptide decreased in a time-dependent manner and approached zero following approximately 8 h incubation period at 37 °C (Fig. 3A,E,F), indicating that nearly all peptides aggregated and formed fibrils. This is because the spin relaxation rate during ^1^H NMR detection is inversely correlated to the overall rotational motion of the molecule^22^, resulting in impaired NMR signals to be observed for very large molecules, such as fibrils. For the Aβmut-GFP, the spectra were unchanged even after a 63.5 h incubation at 37 °C (Fig. 3C), indicating that the monomeric state persists at 37 °C. The spectral intensity of the Aβ-GFP protein decreased by approximately 20% during the first 15.5 h incubation at 37 °C (green line), but not significantly during the subsequent 48 h (blue line, Fig. 3B). These data suggested that the aggregation stopped before fibril formation.

**Figure 4:**
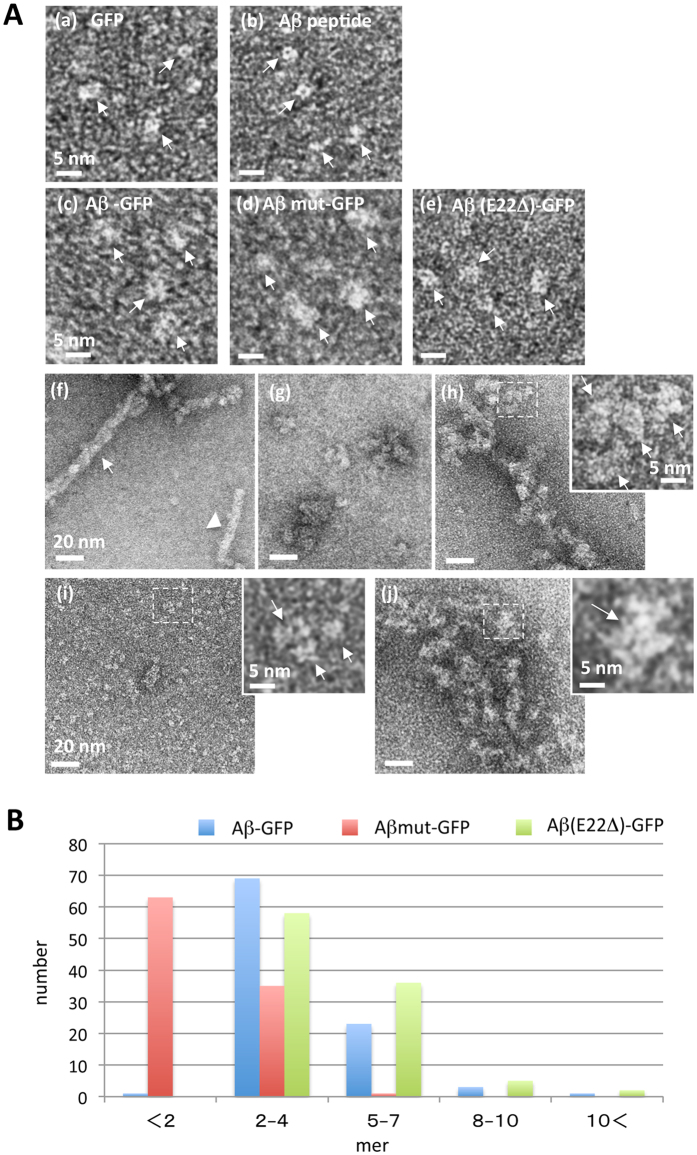
EM analysis of molecular feature of Aβ-GFP fusion proteins. EM images (**A**) and analyses (**B**) of Aβ-GFP fusion proteins. GFP (a), monomeric Aβ peptide (b), Aβ-GFP (c), Aβmut-GFP (d), and Aβ (E22Δ)-GFP (e) are indicated by arrows in each panel. 24 h after incubation at 4 °C (pH8.5), Aβ peptide formed long fibrils (f) but Aβ-GFP (g,h) and Aβ (E22Δ)-GFP (j) formed oligomers with various sizes (g,j) or filamentous-looking aggregates (h). Almost all the Aβmut-GFP remained as small particles in the size of a monomer or a very small oligomer (i) without a clear sign of polymerization. The inset shows a magnified view of the dotted rectangle in (h–j) revealing single units of Aβ-GFP fusion protein oligomers (arrows). Measurement of the area of each unit (**B**) shows that a single unit of polymerized Aβ-GFP and Aβ (E22Δ)-GFP contains two to four molecules but the particles observed with Aβmut-GFP contain single to two molecules (n = 100 units). Scale bars: 5 nm (a–e) and insets), 20 nm (f–j).

Next, we examined the molecular features of each Aβ-GFP fusion protein by negative-stain EM. The images of GFP showed round or rectangular particles ~3–4 nm in size (Fig. 4Aa), consistent with the atomic structure of GFP^23^. Under unpolymerizing conditions for Aβ, the Aβ peptide was observed as smaller, round or elongated particles (Fig. 4Ab), possibly corresponding to single Aβ peptides. In the images obtained for Aβ-GFP, Aβmut-GFP, and Aβ(E22Δ)-GFP, some particles were only slightly larger than GFP, probably corresponding to single fusion proteins (Fig. 4Ac–e). Under the conditions that promote polymerization, the synthetic Aβ peptides formed fibrils of 7.34 ± 0.36 nm (n = 30) in width (arrowhead), or thicker filaments (arrow), which appeared to form following entwining of the fibrils with each other (Fig. 4Af). In contrast, Aβ-GFP, Aβ(E22Δ)-GFP, and Aβmut-GFP did not form regular fibrils. Instead, Aβ-GFP was observed as oligomers of various sizes (Fig. 4Ag) or as filamentous-looking aggregates (Fig. 4Ah), and Aβ(E22Δ)-GFP was also observed as oligomers of various sizes (Fig. 4Aj). Magnified views of the dotted rectangles (inset of Fig. 4Ah,j) reveal that these aggregates are composed of small oligomeric clusters of ~10 nm. We call the single oligomeric cluster as 1 unit (arrows in inset). In the case of Aβmut-GFP, however, these clusters were rarely observed and most of the molecules seemed to be in monomers or very small oligomers (Fig. 4Ai and magnified view of dotted rectangle in i).

We examined how many molecules were present in single units of Aβ-GFP and Aβ(E22Δ)-GFP aggregates, or in a particle observed with Aβmut-GFP. The estimated area of a single Aβ-GFP fusion protein was 13.7 nm^2^. Therefore, the actual measured values of areas for each single unit of Aβ-GFP aggregates were divided by 13.7 nm^2^. Figure 4B showed that the main species of single units of Aβ-GFP and Aβ(E22Δ)-GFP fusion protein oligomers contained 2–4 molecules. The averaged number of molecules in one unit of Aβ(E22Δ)-GFP seemed to be slightly larger than that of Aβ-GFP. The main species of Aβmut-GFP was monomer to dimer, consistent with the idea that this mutation suppresses aggregation of Aβ. With all of the three fusion proteins, some small aggregates were observed, but long fibrils were not formed. Thus, both NMR and EM studies suggest that Aβ-GFP fusion proteins form small oligomers.

### Fluorescence correlation spectroscopy (FCS) analysis of Aβ-GFP fusion protein in living cells

To further confirm the oligomeric state of the Aβ-GFP fusion protein in living cells, we performed FCS analysis on cells expressing each fusion protein as well as on their lysates. First, we examined the properties of each fusion protein in aqueous solution, which were extracted from transfected COS7 cells. Compared to the diffusion constant for the GFP protein (111.0 μm^2^/s), that of the Aβ-GFP fusion protein was significantly lower (74.8 μm^2^/s, Cell lysate in Table 1), indicating that the protein mobility of the fusion protein was significantly decreased. The Aβmut-GFP protein showed an intermediate diffusion constant (86.6 μm^2^/s) between that of GFP and Aβ-GFP. Since the estimated molecular weight of single Aβ-GFP fusion protein is not markedly different from that of GFP (33 kDa vs 27 kDa), the decreased diffusion mobility observed with the Aβ-GFP fusion protein suggested that some molecular complex, presumably oligomers composed of several Aβ-GFP fusion protein molecules, are formed within cells. Interestingly, the count per molecule (CPM) value of the Aβ-GFP fusion protein was decreased compared to that of GFP, whereas that of Aβmut-GFP showed similar value to that of GFP, suggesting that an increase in fluorescent intensity of a particle does not simply occur, even if the fusion protein forms oligomers (see the “Discussion” section).

We applied this observation directly to living COS7 cells and the results were similar to those obtained in aqueous condition (Live cell in Table 1). Because of restricted diffusion in the cell, autocorrelation functions were fitted using a two-component diffusion model for this analysis. The diffusion constant of Aβ-GFP in both the cytoplasm and nucleus was significantly lower than that of GFP, again suggesting decreased diffusion mobility due to the formation of larger molecular complex compared to GFP (estimated Mw; 27 kDa in GFP vs 77 kDa in Aβ-GFP. See the “Methods” section for calculation). The two-component diffusion model analysis applied for living cells also showed that remarkable decrease of fast component fraction in Aβ-GFP expressing cells (84%), compared to both GFP and Aβmut-GFP (around 95%). This suggests that interactions between the Aβ-GFP protein and other intracellular species may be increased and/or the amount of large soluble aggregates formed by the Aβ-GFP protein may be increased within cells. The CPM value from cells expressing Aβ-GFP was decreased compared to that of GFP and Aβmut-GFP, suggesting that fluorescence in the large soluble aggregates/oligomers may be quenched (see the “Discussion” section for further explanation). We have also applied this observation to examine the molecular dynamics of Aβ(E22Δ)-GFP in cell lysate and in living cells. In both conditions, this mutant form showed significantly slower mobility than GFP, but the mobility was not significantly different from that of the wild-type Aβ-GFP in any conditions. These results suggest that the E22Δ mutation also causes the formation of large protein complex similar to the wild-type Aβ-GFP. These FCS data confirmed that our fusion proteins generated by a long-linker sequence showed robust fluorescence and can be used to monitor the molecular dynamics of Aβ containing various types of mutations.

### Expression of Aβ-GFP fusion proteins in transgenic *C. elegans*

**Figure 5:**
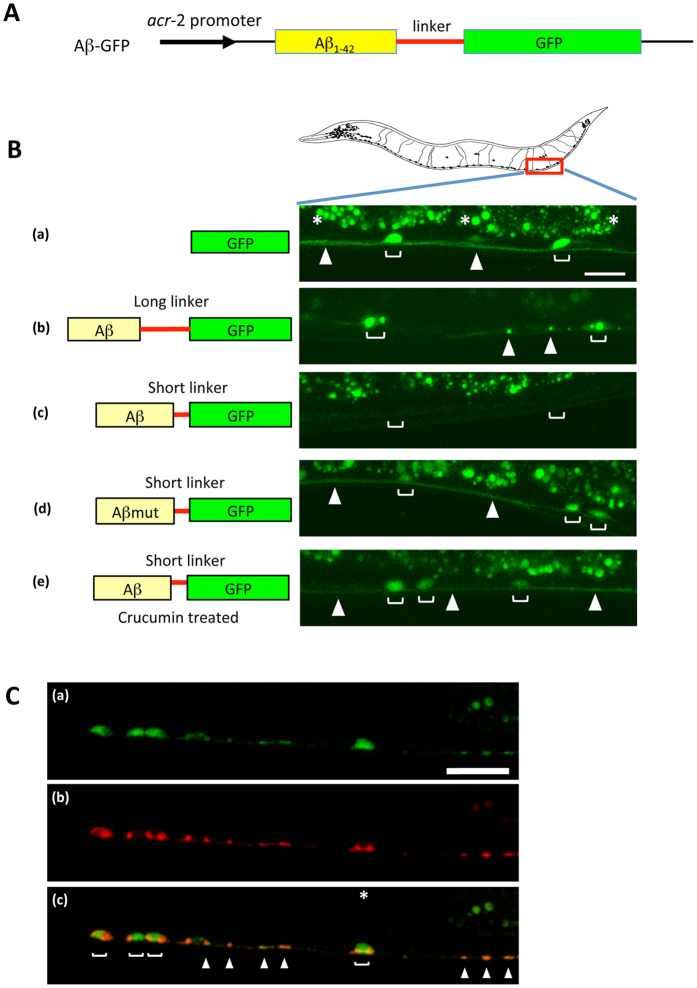
Expression of Aβ-GFP fusion proteins in *C. elegans*. (**A**) Schematic representation of the Aβ-GFP fusion construct. (**B**) GFP fluorescence in the cholinergic motor neurons of Aβ-GFP transgenic *C. elegans*. The left illustration depicts the expressed proteins shown in the right pictures. The right pictures show the expression patterns of fusion proteins in *C. elegans*. (a) GFP, (b) Aβ-GFP with a long-linker, (c) Aβ-GFP with a short-linker, (d) Aβmut-GFP with a short-linker, and (e) crucumin treatment of animals bearing a short linker protein. Blankets indicate the cell bodies of neurons and arrowheads indicate the axon in the ventral nerve cord. Asterisks indicate the autofluorescence from the intestine. The long-linker has 14 amino acids and the short-linker has only 2 amino acids sequences. Cells expressing the short-linker Aβ-GFP protein did not show fluorescence (c) but the long-linker one and Aβmut-GFP showed bright fluorescence (b,d). Short-linker Aβ-GFP transgenic *C. elegans* were treated with curcumin, which induces Aβ disaggregation. Disappeared fluorescence was recovered after treatment with curcumin (e). Scale bar: 10 μm. (**C**) Localization of the Aβ-GFP fusion protein at the presynaptic regions. Aβ-GFP (a) and presynaptic protein SNB-1 fused with mCherry (b) were simultaneously expressed in cholinergic neurons. Several GFP puncta were co-localized with SNB-1 on the axon (c) suggesting that the fusion protein may be strongly accumulated at synaptic sites. Scale bar: 10 μm.

Both the *in vitro* analyses of the molecular state of Aβ-GFP fusion proteins and the *in vivo* analyses of living cultured cells suggested that the fusion proteins probably exist as oligomers. These results also indicated that the fluorescence of the fusion proteins can be altered dependent on their aggregation properties when a short-linker is used. To examine whether these phenomena can also be observed in neuronal cells of a living animal, we expressed our fusion proteins in *C. elegans* neurons and observed their dynamics *in vivo*. A schematic representation of the Aβ-GFP fusion construct used for transgenic *C. elegans* strains is shown in Fig. 5A. Aβ-GFP was specifically expressed in the cholinergic neurons by the *acr*-2 promoter. GFP fluorescence was detected steadily inside of the neurons in GFP transgenic animals (Fig. 5Ba). In transgenic animals expressing long-linker Aβ-GFP, GFP fluorescence was observed in both the cell bodies and their neurites, but showed accumulated or aggregated expression patterns of the fusion protein (Fig. 5Bb). However, GFP fluorescence was absent in the short-linker Aβ-GFP transgenic worms (Fig. 5Bc), which is similar to the expression patterns observed in COS7 cells and rat hippocampus primary neurons (Fig. 2).

We also wondered whether the fluorescence intensities in transgenic animals expressing short-linker Aβ-GFP reflect the aggregation properties of fusion proteins. To examine this, we expressed Aβmut-GFP fusion protein with the short-linker, and GFP fluorescence was clearly and uniformly detected in the neuronal cells of Aβmut-GFP transgenic worms (Fig. 5Bd). This finding indicates that non-fibril and soluble forms of Aβ do not affect the folding of GFP and that GFP fluorescence can be observed in living neurons if aggregation of the fusion protein is inhibited.

Therefore we examine whether these phenomena could be used to screen for substance that inhibit Aβ aggregation. It is known that curcumin can inhibit polymerization of Aβ. Thus we added it to the culture medium and the molecular state of short-linker forms of Aβ-GFP was observed in transgenic worms. In the animals reared on plates containing curcumin, bright and uniform GFP fluorescence was observed in both cell bodies and neurites, similar to animals expressing the Aβmut-GFP protein (Fig. 5Be). These findings indicated that the inhibition of Aβ aggregation induced by curcumin results in the recovery of GFP fluorescence.

This fusion protein can be also used to examine the subcellular localization of Aβ protein (Fig. 5C). The presynaptic VAMP2 protein (SNB-1 in *C. elegans*) was fused to mCherry and simultaneously expressed with the long-linker Aβ-GFP fusion protein, under the control of the same promoter. Several strong accumulations of the Aβ-GFP fusion protein correlated well with the position of RFP localization, meaning that the fusion protein tended to accumulate at the synaptic regions when the protein is expressed in presynaptic neurons.

### Effect of Aβ-GFP oligomers on survival rate of COS7 cells

**Figure 6:**
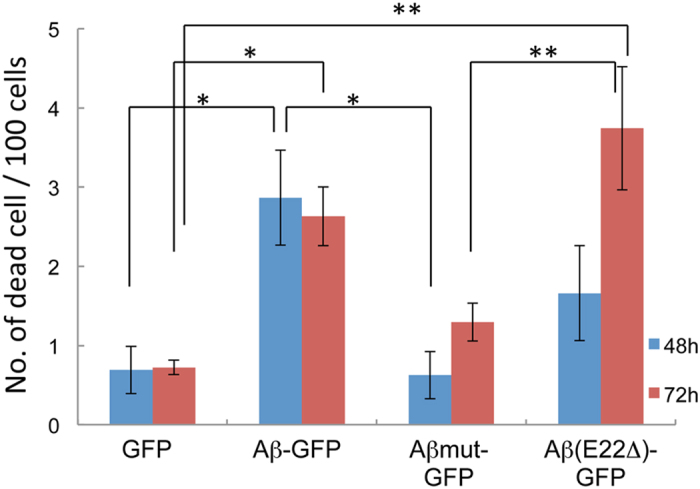
Effect of Aβ-GFP fusion proteins on the survival of COS7 cells. The numbers of dead COS7 cells were counted 48 h (blue) and 72 h (red) after transfection with plasmids encoding GFP, Aβ-GFP, Aβmut-GFP, or Aβ (E22Δ)-GFP. The number of dead cells increased significantly in Aβ-GFP and Aβ (E22Δ)-GFP transfected cells compared with the Aβmut-GFP and GFP control transfected cells. The data represents the mean ± SEM (100 cells), *p < 0.05, **p < 0.001 by one-way ANOVA.

To examine whether the Aβ-GFP fusion protein caused cellular toxicity in living cells, we measured cell death ratios in COS7 cells transfected with each Aβ-GFP fusion plasmid or the GFP plasmid (Fig. 6). Compared with cells expressing GFP, the ratios of dead cells significantly increased in both Aβ-GFP and Aβ (E22Δ)-GFP transfected COS7 cells until 72 h after transfection, but it was not changed in Aβmut-GFP expressing cells. These results indicate that Aβ-GFP and Aβ (E22Δ)-GFP oligomer may cause cellular toxicities like wild-type Aβ oligomers.

## Discussion

The intracellular accumulation of Aβ_1-42_ has been proposed as an event responsible for early pathogenesis of AD. Especially, Aβ oligomer has been the subject of much attention as a target for studying the pathophysiological role of AD^24^,^25^, because it has been proposed to be a key mediator of cognitive decline in AD^11^. In this study, we developed new cellular and animal models of AD, which showed an accumulation of small sized Aβ oligomers inside of cells. This molecular state can be achieved by fusing Aβ and GFP, and this method can be used to visualize the molecular dynamics of Aβ in living cells by arranging the linker sequence between Aβ and GFP.

Previous report using yeast lysate that expresses Aβ-GFP fusion proteins suggested that Aβ_1-40_-GFP and the Aβ-GFP mutant that contains substitution Ile 41 to Glu and Ala 42 to Pro are less prone to aggregation and a portion of those fusion proteins exhibit soluble and non-aggregated forms, but Aβ_1-42_-GFP exhibit insoluble aggregate only^26^. We confirmed the molecular features of GFP-fused Aβ proteins through several strategies. In NMR experiments, we started the measurement for all samples under monomer conditions and the same concentration of proteins. Previous findings indicated that GFP is stable at pH 6–10^27^,^28^ and that NaOH does not affect the conformational, tinctorial, morphological, and physiological functions of Aβ^29^. Therefore, our methods to form monomers should not affect the protein properties of Aβ-GFP fusion proteins. Our results indicated that the synthetic peptide formed fibrils within 8 h incubation period, however, the multimerization of Aβ-GFP proteins stopped before 15.5 h, consequently they could not form fibrils and remained as oligomers. In the EM experiments, the synthetic peptides formed long fibrils, but Aβ-GFP formed oligomers consisting of mainly 2–4 molecules, which did not assemble further into fibrils or large aggregates. These results were consistent with the NMR results and showed that Aβ-GFP form oligomers *in vitro*. The fusion protein is composed of a 27 kDa GFP component and a 4.5 kDa Aβ, thus a GFP molecule is much larger than the Aβ molecule. Therefore, aggregation of Aβ might be sterically hindered by GFP and, as a result, Aβ-GFP fusion protein could form only oligomers.

FCS analysis also suggested that the same molecular states of Aβ-GFP fusion proteins exist in living cells. In cultured living cells, the estimated molecular size of Aβ-GFP calculated from the diffusion constant was 77 kDa in the cytoplasm and 64 kDa in the nucleus. The larger molecular sizes probably result from either the formation of oligomers or molecular complexes with intracellular proteins. Contrary to the slow diffusion mobility of the Aβ-GFP fusion proteins, their CPM values, which refer to fluorescence intensities per single particle, were smaller than that of GFP as well as a non-aggregating mutant, Aβmut-GFP (Table 1). These results suggest that homo-oligomeric species of Aβ-GFP may emit low fluorescence intensity because of quenching of GFP fluorescence. By fusing Aβ_1-42_ to the N-terminus of GFP, the folding properties of GFP could be altered, and only a small fraction of the fusion proteins can express fluorescence^17^,^18^. Due to this fusion protein’s nature, the CPM values of the Aβ-GFP fusion protein may not appear to correspond to the multimerization state of the fusion protein. Based on this argument, we focused on the values of diffusion constant and estimate that the major forms of oligomers of the Aβ-GFP protein may be trimers to tetramers in live cells. In contrast, Aβmut-GFP showed a fraction of the fast component similar to that of GFP, but the estimates molecular weight (41 kDa) was between that of a monomer and a dimer, suggesting that the fusion protein probably exists as a mixture of monomers and dimers. The molecular state of the Aβ(E22Δ)-GFP mutant did not show a clear difference in molecular mobility from the wild-type Aβ-GFP, although it causes AD and shows an age-dependent intraneuronal accumulation of oligomers^16^. We believe that, however, our methods enable us to understand the intracellular dynamics of these kinds of mutant proteins of Aβ. Further investigations of appropriate linkers to generate Aβ-GFP with a less quenching property will clearly improve detection sensitivity of the Aβ oligomers in live cells using FCS. In COS7 cells transfected with the Aβ-GFP plasmid, almost all Aβ-GFP fusion proteins were labeled with the 11A1 antibody, which recognizes oligomeric Aβ specifically^20^. The decrease of the fast fraction in Aβ-GFP (84% and 83%) also means that larger complexes probably exist in living cells. Taken together, these data suggested that Aβ-GFP fusion proteins formed small oligomers inside cells and that this fusion protein is a new useful tool for assessing the intracellular function and toxicity of the Aβ oligomers.

Previously, it was reported that insoluble aggregates of N-terminal fusion partners with GFP attenuated the fluorescence of GFP^17^. A linker was constructed using 12 amino acids to make a reporter construct for evaluating protein folding, which was designed to avoid large bulky hydrophobic residues. Wurth *et al.*^18^ used the same construct to make a fusion protein composed of a N-terminal GFP fused to C-terminal end of Aβ_1-42_. In this case, the wild type Aβ-GFP fusion protein did not fluoresce in *E. coli* whereas strong fluorescence was observed in the mutated Aβ-GFP fusions containing substitutions in the hydrophobic region responsible to aggregation of Aβ. Nair *et al.*^26^ also reported using mutant Aβ-GFP fusion constructs with which aggregation is reduced that the fluorescence intensity in yeast reflects the aggregation state of Aβ. In the current study, we inserted 14 random amino acids as a linker sequence between Aβ and GFP (long-linker), and succeeded in developing new Aβ-GFP fusion proteins that fluoresce even when the wild type Aβ is aggregated. We do not fully understand the exact reasons why this linker sequence can generate stable fluorescence in an aggregated condition. Using this construct, however, it is possible to observe the molecular dynamics of Aβ-GFP oligomers in living cells. There are some reports suggesting that Aβ provokes ER stress and oxidative stress and induces cellular toxicities^30^,^31^,^32^,^33^,^34^,^35^. In particular, the E22Δ mutant caused ER stress, which induced apoptosis in HEK293 cells^36^. We also found that both Aβ-GFP and Aβ (E22Δ)-GFP caused increased cell death in transfected COS7 cells. These findings indicated that GFP-tagged Aβ fusion proteins may have similar physiological properties as wild type Aβ suggesting that they are quite applicable for examining “oligomer hypothesis” in AD. From our EM and FCS results, the molecular states of wild type Aβ-GFP and Aβ(E22Δ)-GFP are not greatly different from each other. Therefore there is a possibility that larger aggregates have a potential to induce strong toxicity compared to smaller aggregates, but the toxicity of these fusion proteins against cells may not depend solely on the aggregation sizes of Aβ. Although the difference in the structures of these fusion proteins are not known, there might be unknown interactive factors with Aβ oligomers that induce the toxicity inside cells.

To evaluate the property of our fusion proteins in living animals, we used *C. elegans* as an experimental model and observed *in vivo* Aβ dynamics. Although invertebrate is phylogenetically far removed from mammals, *C. elegans* possesses several genes homologous to the human AD- related genes such as nicastrin^37^, presenilin^38^,^39^, APH-1^40^ and neprilysin^41^. In addition to these genetic relationships, over expression of Aβ exhibits an increased level of reactive oxygen species (ROS) in *C. elegans*^42^ similar to those observed in AD patients. In *C. elegans* neurons, we confirm that our fusion proteins showed fluorescence properties quite similar to those in mammalian cells including rat primary cultured hippocampal neurons and COS7 cells, i.e., the protein with the short linker decreases its fluorescence when it aggregates, whereas the long linker retains fluorescence in spite of its aggregation. Therefore, *C. elegans*, as well as mammalian models, is an useful tool to investigate the basic pathogenesis of AD. Using this model system, we can perform both the genetic and chemical screening for endogenous or exogenous factors that are possible to regulate the Aβ aggregation in live animals. For example, the curcumin experiment in short- linker Aβ-GFP transgenic worms revealed that it is possible to monitor the effect of drugs against deposition and disaggregation of Aβ directly in living animals, suggesting that our new fusion proteins are useful tools for screening candidate synthetic chemical and naturally occurring products against AD in living neuron.

## Methods

### Plasmids construction

To generate the Aβ_1-42_-GFP expression vector, we amplified Aβ_1-42_ from a plasmid containing the human APP coding sequence (DNAFORM, clone ID:100068486) with a 5′ primer including a *Hind*III site and 3′ primer including a *Sal*I site. The resultant PCR fragment was subcloned into a modified pEGFP-N1 vector (Takara BIO Inc, Shiga, Japan) containing a chicken β-actin promoter^43^ to generate the pAct-Aβ_1-42_-GFP plasmid. Expression vectors encoding Aβ_1-42_-GFP proteins with short-linkers, which include 0, 2(LE), or 3(LET) amino acids of the linker sequence between Aβ_1-42_ and GFP, were generated by deleting the linker sequence from the pAct-Aβ_1-42_-GFP plasmid.

Mutant forms of Aβ-GFP were generated using GeneArt^®^Site-Directed Mutagenesis System (Life Technologies) with the pAct-Aβ_1-42_-GFP plasmid serving as a template. The Aβmut-GFP is a mutant generated by substituting Phe19 with Ser and Leu34 with Pro. The Aβ_1-42_ (E22Δ)-GFP is a deletion mutant constructed by removing glutamate-22 of Aβ_1-42_ sequence from the pAct-Aβ_1-42_-GFP plasmid.

To express Aβ-GFP fusion genes in *C. elegans*, the human Aβ_1-42_ coding sequence was inserted into the Fire’s *C. elegans* GFP expression vector (a kind gift from A. Fire). A 3.0 kb upstream region of the *acr-2* gene was used to specifically express the fusion proteins in cholinergic motor neurons. The same promoter region was inserted into the mCherry vector to generate a *Pacr-2: snb-1: mCherry* fusion construct. The *C. elegans snb-1* cDNA fragment was amplified by RT-PCR and was subcloned in frame into the *Pacr2: mCherry* vector. All plasmid DNAs were sequenced, and the sequences are available on request.

### Generation of transgenic *C. elegans*

Transgenic worms were generated using standard microinjection methods for *C. elegans*. The pbLH98 [*lin-15(+)*] or P*acr-2*:: RFP plasmid was used as a co-injection marker at 50 ng/μl. Each fusion plasmid was injected at 10–25 ng/μl. At least 3 independent stable transgenic lines were used in the experiments performed in this study.

### Cell culture and transfection

All experimental procedures performed on animals were carried out in accordance with the approved guidelines in ethical permit approved by the Institutional Animal Care and Use Committee of the National Institute of Advanced Industrial Science and Technology (Permission No. 2014-143) and in accordance with the Law No. 105 passed by and the Notification No. 6 released by the Japanese Government.

Primary cultures were prepared from the hippocampi of embryonic day 17 Wister rats (Nihon SLC, Shizuoka, Japan). Briefly, embryos were removed by cesarean sectioning, after which hippocampi were isolated and digested in 1 U/ml papain (Worthington Biochemical corporation, NJ, USA) in HEPES-buffered Hank’s Balanced Salt Solution containing cysteine and BSA (1 mg/ml each) at 37 °C for 12 min. Dissociated cells were plated on poly-L-lysine coated glass cover slips at a density of 30,000 cells/ well in a 6-well plate in plating medium containing Dulbecco’s Modified Eagle Medium (DMEM, Wako, Osaka, Japan) and 10% fetal bovine serum (FBS, Gibco, NY, USA). Two hours after plating, the medium was changed to Neurobasal^®^ Medium (Invitrogen, CA, USA) containing B27 supplement (Invitrogen) and 0.5 mM L-glutamine.

COS7 cells were cultured in DMEM containing 10% FBS at 37 °C in a humidified atmosphere containing 5% CO_2_/95% air. For transfection of plasmid DNAs, cells were plated at a density of 15,000 cells/cm^2^ in a 6-well plate.

COS7 cells and 4DIV primary hippocampal neurons were transfected with plasmids using Lipofectamine 2000 (Invitrogen), as described previously^44^. Dead cells were counted 48 h and 72 h after transfection by staining the living COS7 cells with DAPI. The DAPI solution (Dojindo, Kumamoto, Japan) was added to the medium of each transfected cells and incubated for 20 min. After washed by PBS, five images were taken from each culture dish, and cells stained by DAPI were counted as dead. The total number of cells in images were also counted and used to calculate the number of dead cells per 100 cells. Statistical analysis was performed by one-way analysis of variance (ANOVA).

### Immunohistochemistry and imaging of cells

To confirm the expression of each Aβ-GFP fusion protein, transfected COS7 cells and primary neurons were immunostained with an anti-β amyloid (6E10) antibody (Covance, WI, USA) or 11A1 antibody (IBL, Gunma, Japan). Twenty-four hours after transfection, cells were fixed with 4% paraformaldehyde and 4% sucrose in PBS for 15 min at room temperature. Then, the cells were washed with PBS and permeabilized for 5 min in 0.25% Triton X-100. After washing the cells in PBS, they were blocked in 3% normal goat serum (Vector Laboratories, Inc., CA, USA) for 30 min and incubated with the 6E10 antibody (×800 dilution) or 11A1 antibody (×50 dilution) overnight at 4 °C. Each protein was visualized with an Alexa 568 conjugated secondary antibody (Invitrogen), and the nuclei were labeled with DAPI (Vector Laboratories, Inc.). Cell imaging was performed using Olympus FluoView 1000 (Olympus, Tokyo, Japan) and Nikon A1R (Nikon, Tokyo, Japan) confocal laser scanning microscopes. Each image was obtained at a resolution of 1020 × 1020 pixels. To quantify fluorescence intensities, the confocal scanning settings of pinhole, laser power, brightness, and contrast were held constant for all images. The fluorescence intensities of cell bodies were measured using Image J software (NIH, Bethesda, MD, USA). Statistical analysis of fluorescence intensities was performed by the Kruskal-Wallis test (n = 10–14 cells each).

Time laps imaging was performed using COS7 cells transfected with Aβ-GFP plasmid containing long-linker. Eighteen hours after transfection, fluorescent images of cells were taken every 10 min for 24 h in a humidified atmosphere containing 5% CO_2_/ 95% air at 37 °C using a × 20 objective lens (KEYENCE corporation, Osaka, Japan).

### Curcumin treatment of Aβ-GFP transgenic *C. elegans*

Curcumin were dissolved in ethanol, and 100 μl of 3 mM solution was spread onto NGM plates. Young-adult transgenic worms expressing short-linker Aβ-GFP protein were placed on the curcumin plates, and the fluorescence of their progeny was examined.

### Purification of the Aβ-GFP fusion protein

Purifications of recombinant proteins were performed according to the manufacturer’s instructions (New England BioLabs, MA, USA) and Chong *et al.*^45^. Briefly, the coding sequences of Aβ-GFP, Aβmut-GFP, and GFP were cloned into the pTXB1 vector (New England BioLabs) and the resulting plasmid DNAs were transformed into *E. coli* BL21 cells. The cells were grown in LB media at 37 °C until the culture reached an OD_600_ _nm_ of 0.5, and then the cells expressed the fusion protein by adding of 0.2 mM IPTG and incubated at 30 °C for 4 h. Cells were harvested and resuspended in Tris buffered solution (buffer A: 20 mM Tris-HCl, pH 8.5, 300 mM NaCl and 10% glycerol). After adding 0.002% CHAPS, 0.05 mM EDTA (pH 8.0) and 0.1 mM PMSF, the cell suspensions were incubated for 30 min at 4 °C and then ultrasonic disruption were performed on ice, using a BRANSON SONIFIRE 250. The lysed cell suspensions were centrifuged at 9600 × g for 20 min at 4 °C, after which the supernatants were loaded onto equilibrated Chitin beads (New England BioLabs) and incubated for 1 h on rotator at 4 °C. The beads were loaded in a column and washed with buffer A, and then the fusion proteins were eluted by Tris buffered solution (buffer B: buffer A containing 50 mM DTT) for 16 h at room temperature. The concentrations of proteins were measured using the BCA protein assay kit (Pierce). Purified fusion proteins were stored at −80 °C until use.

### NMR analysis

To produce the monomeric Aβ-GFP and Aβmut-GFP, 1 N NaOH were added to the purified protein solution until the pH reached 10-11, and then the protein solution was sonicated for 1 min^21^. Synthetic Aβ peptide (Peptide Institute, Inc. Osaka, Japan) was predissolved in 10 mM NaOH solution at a concentration of 500 μM. Then, the buffer of each protein was displaced to 20 mM deuterated Tris-HCl (d_11_, 99%; CDN isotope, QC, Canada) at pH 7.2 containing 0.05 mM NaN_3_ by ultrafiltration. After centrifugation at 15,000 × g for 5 min, the solutions were kept at 4 °C. Purified GFP was also subjected to the above process. Immediately before the NMR measurements, solutions of 25 μM protein/peptide were dissolved in 20 mM deuterated Tris-HCl (pH 7.2) containing 0.1 mM sodium 2,2-dimethyl-2-silapentane-5-sulfonate and 5% D_2_O (Cambridge Isotope, MA, USA).

NMR measurements were performed on a Bruker Avance III-500 spectrometer at 20 °C. Water suppression was achieved by the WATERGATE method^46^. In addition to the monomeric proteins/peptides, those incubated at 37 °C for 15.5 h or 50–63.5 h were subjected to the measurements. Also, successive NMR measurements of the Aβ peptide solution at 37 °C for a real-time monitoring of aggregation were carried out.

### Electron microscopic analysis

Monomeric and polymerized fusion proteins were observed by EM. Monomeric fusion proteins were prepared as described above. Polymerizations were performed by incubating the monomers at 4 °C for 24 h in buffer A. Synthetic peptides (generous gifts from Dr. Miyagishi, AIST) were pre-dissolved in NH_3_ and polymerized in buffer A at a ×1000 dilution. For the EM observations, each sample was diluted to a final concentration of 0.2 μM.

Small aliquots (5 μl) of samples were deposited onto carbon coated EM grids and negatively stained with 1% uranyl acetate, as described^47^. After blotting away the excess solution and air-drying, observations were performed using a Tecnai F20 EM (FEI) operated at 120 kV. The images were recorded with an ORIUS SC600 slow-scan CCD camera at a magnification of ×80,000.

Length, diameter, and area measurement were performed using Image J software. The areas were used to evaluating how many single molecules were polymerized to form a single unit of Aβ-GFP fusion protein oligomers. The observed areas of the oligomers were divided by that of the monomeric Aβ-GFP fusion protein. The area of a single GFP protein is ~12 nm^2^, based on the dimensions of the atomic structure of a GFP molecule (~3 nm × 4 nm)^23^, and the observed images agreed with the prediction (see Results). Since the ratio of the molecular weights of GFP : Aβ: Aβ-GFP (including the linker sequence) is ~27: 5: 33, we estimated that the ratio of the area would be roughly 9: 2.9: 10.3, so that the area of a monomeric Aβ-GFP is ~13.7 nm^2^.

### FCS measurement

FCS measurements were performed as described previously^48^,^49^,^50^. For the *in vivo* analysis of living cells, COS7 cells were cultured on 35 mm glass base dish (AGC Techno Glass, Shizuoka, Japan), and transfected with GFP or Aβ-GFP plasmids at 17–27 h before analysis. FCS measurements in live cells were performed for both the cytoplasmic and nucleus regions in the same transfected cells, using an LSM 510 META confocal microscopy equipped with ConfoCor3 (Carl Zeiss, Jena, Germany). GFP was excited at 488 nm using an Argon ion laser through a C-Apochromat 40 × 1.4NA Korr. UV-VIS-IR water immersion objective. For the analysis of cell lysates, transfected COS7 cells were once washed in ice cold PBS and lysed in CelLytic M (Sigma-Aldrich, MO, USA) containing cOmplete Protease Inhibitor cocktail (Roche Diagnostics, Basel, Germany). After a 15 min incubation on shaker at 4 °C, the cells were harvested and their supernatants were recovered after centrifugation (12,000 × g) for 15 min. FCS measurements from cell lysates (aqueous solution) were performed using the Hamamatsu FCS Compact system (Hamamatsu Photonics, Shizuoka, Japan). Fluorescence autocorrelation functions in cell lysates were measured for 30 sec and analyzed using an one-component model, while those in living cells were measured for 60 sec and fitted using a two-component diffusion model. In both analyses, the structure parameters were determined using rhodamine 6 G (Rh6G) and were set from 4 to 6. The diffusion constant of fusion proteins (*D*_*sample*_) was calculated as follows:


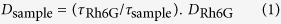


where *τ*_Rh6G_ and *τ*_sample_ are the diffusion time of Rh6G and the sample, respectively, and *D*_Rh6G_ is the given diffusion constant of Rh6G (414 μm^2^/s). CPM values were normalized using that of GFP observed in the same experimental set. The molecular weight (*MW*) of the fusion proteins was calculated using following equation modified from Stokes-Einstein relation:


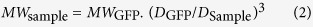


## Additional Information

**How to cite this article**: Ochiishi, T. *et al.* Development of new fusion proteins for visualizing amyloid-β oligomers *in vivo*. *Sci. Rep.* **6**, 22712; doi: 10.1038/srep22712 (2016).

## Supplementary Material

Supplementary Information

Supplementary Figure S2

## Figures and Tables

**Table 1:**
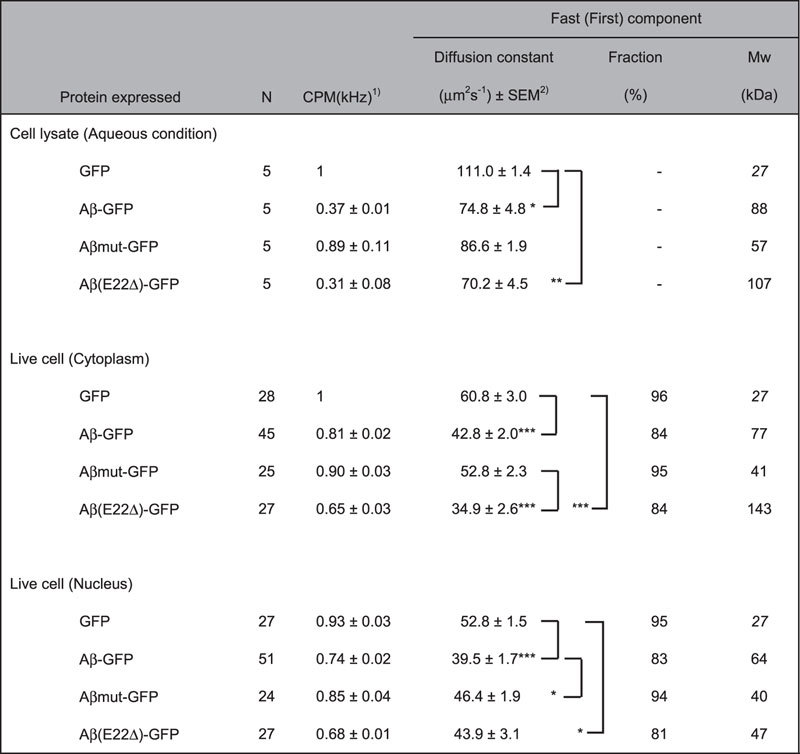
FCS analysis of Aβ-GFP proteins in living cells.

^1^CPM values from lysate samples are normalized by that of GFP, and live-cell CPMs are normalized by that of GFP in cytoplasm.

^2^Kruskal-Wallis and post-hock tests are performed among each condition. Only the significant differences are shown: ***P < 0.001, **P < 0.01, *P < 0.05.
