# Supplementary material for: Development of new fusion proteins for visualizing amyloid-β oligomers in vivo
Source: Sci Rep. 2016 Mar 16;6:22712. doi: 10.1038/srep22712 (PMC4793674; doi:10.1038/srep22712)
Supplement: Supplementary Information [file srep22712-s1.pdf]

Supplementary Information

Development of new fusion proteins for visualizing amyloid-β oligomers *in vivo*

Tomoyo Ochiishi, Motomichi Doi, Kazuhiko Yamasaki, Keiko Hirose, Akira Kitamura, Takao Urabe, Nobutaka Hattori, Masataka Kinjo, Tatsuhiko Ebihara, Hideki Shimura

Supplementary Figure S1

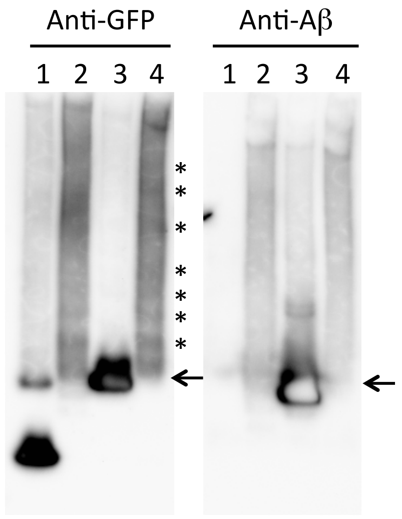

Supplementary Figure S1: Aβ-GFP protein exists as oligomer in living cells.
COS7 cells were transfected with GFP (lane 1), Aβ-GFP (lane 2), Aβmut-GFP (lane 3), or Aβ (E22Δ)-GFP (lane 4) plasmid DNAs and extracted protein samples from those cells were separated by a native-PAGE and immunoblotted by either anti-GFP or anti-Aβ antibody. Single strong band in the Aβmut-GFP means that most of this protein exists as monomer in the cells (black arrows). On the other hands, smear and wide-ranged ladder-like signals were detected in the lanes from Aβ-GFP and Aβ (E22Δ)-GFP, suggesting that these two proteins form several sizes of oligomers in the cells (asterisks in the anti-GFP membrane).

Supplementary Figure S2: Dynamic movement of Aβ-GFP fusion proteins in living cell (video).

COS7 cells were transfected with Aβ-GFP plasmid and time-laps imaging was performed. Aβ-GFP fusion proteins gradually aggregated in a time-dependent manner.

Supplementary Methods:

Immunoblotting

COS7 cells were transfected with each plasmid DNA as described above. 48 hours after transfection, cells were washed twice in ice-cold PBS buffer and were lysed in SDS-free lysis buffer (25 mM Tris-HCl (pH 7.5), 100 mM NaCl, 2 mM EDTA, 0.5% Triton X-100, 1 mM PMSF). Lysates were cleared by a 10-min centrifugation at maximum speed. Equal amounts of protein samples (20 μl/lane) were separated on polyacrylamide gels (5-20% gradient gel, Wako), transferred to a PVDF membrane, and blocked with 5% non-fat dry milk in PBS with 0.05% Tween-20 (PBST). Primary antibodies were diluted in PBST buffer as follows: 1;10,000 for anti-GFP (Clontech, CA, USA), 1:3,000 for anti- β amyloid (6E10, Covance), and membranes were incubated at 4ºC for overnight. After washing by PBST, membranes were incubated with HRP-labeled secondary antibodies (Jackson ImmunoResearch, PA, USA) for 1h at RT, and treated with an ImmunoStar LD Chemiluminescence reagent (Wako). Images were captured on a ChemiDoc XRS system (Bio-Rad, CA, USA).
